# A Retrospect of Christmas

**Published:** 1887-02-26

**Authors:** 


### A RETROSPECT OF CHRISTMAS.

(The following lines were written by a patient in Friedenfels Home, St. Leonards.)

'Tis true we were invalids—most of us suffering ;
Shut out, it might seem, from the pleasures of life ;
All of us absent from home and belongings,
From mother or father, or children or wife.
But never was sadness, nor sorrow, nor blindness,
On which cheering rays of light did not fall ;
And for us, deeds of loving, practical kindness
Made the Christmas at Friedenfels happy for all.

Each patient on rising saw something surprising,
And found in the parcel, he opened with zest,
A gift from the matron, or founder, or patron,
Just suited for ailments of EAR, THROAT, or CHEST.

When dinner-time came, all inmates were present
(Though two had but risen from bed for a while) ;
The doctor presided in true genial fashion,
And carved for us all in quite masterly style,
A whisper went round—"Matron hoped that we should
Keep our troublesome coughs as quiet as we could";
All anxious to please her, each man did his best,
And, mid silence unusual, we welcomed our guest—
KING TURKEY ! ! ! ! !

Not a sound was heard, not a croaking note
From asthmatical chest or phthisical throat
Disturbed the reign of King Turkey.

And if ever a bird, foreseeing its destiny,
Had gobbled and stuffed to glorious immensity ;
If ever a doctor who carved scientifically ;
If ever a matron who ruled—say—pontifically ;
(Wisely and firmly, yet, oh, so pacifically)
If ever a cook who could "plump up" and season,
And do all any bird could expect, within reason ;
If ever a housemaid who earned a good name
(For though "run off her legs," she looked pleased, all the same) ;—
All these assisted, as everyone tells,
To make Christmas happy at Friedenfels.

Plum puddings, mince pies, though second in favour,
Were pronounced to be of most excellent flavour.
Of toasts there were none, unless we can can find
Exception in one of a medical kind :
Quoth Matron to Doctor : "Is Christmas expected
The order of daily procedure to spoil ?"
"By no means," cries Doctor ; "pray wind up, as usual,
*With a spoonful of luscious cod-liver oil !*"

There's lots more to tell, and I love retrospection,
But Matron declares my pen I must stay ;
Simply adding that visits of private inspection
May be made to our FRIEDENFELS HOME any day.

